# The DnaK Chaperone System Buffers the Fitness Cost of Antibiotic Resistance Mutations in Mycobacteria

**DOI:** 10.1128/mBio.00123-21

**Published:** 2021-03-30

**Authors:** Allison Fay, John Philip, Priya Saha, Ronald C. Hendrickson, Michael S. Glickman, Kristin Burns-Huang

**Affiliations:** a Immunology Program, Sloan Kettering Institute, Memorial Sloan Kettering Cancer Center, New York, New York, USA; b Microchemistry and Proteomics Core Facility, Sloan Kettering Institute, Memorial Sloan Kettering Cancer Center, New York, New York, USA; c Department of Microbiology and Immunology, Weill Cornell Medicine, New York, New York, USA; IDM/University of Cape Town; New York University School of Medicine

**Keywords:** DnaK, antimicrobial resistance, mycobacteria, protein chaperone

## Abstract

Chaperones aid in protein folding and maintenance of protein integrity. In doing so, they have the unique ability to directly stabilize resistance-conferring amino acid substitutions in drug targets and to counter the stress imparted by these substitutions, thus supporting heritable antimicrobial resistance (AMR). We asked whether chaperones support AMR in Mycobacterium smegmatis, a saprophytic model of Mycobacterium tuberculosis, the causative agent of tuberculosis (TB). We show that DnaK associates with many drug targets and that DnaK associates more with AMR-conferring mutant RNA polymerase (RNAP) than with wild-type RNAP. In addition, frequency-of-resistance (FOR) and fitness studies reveal that the DnaK system of chaperones supports AMR in antimicrobial targets in mycobacteria, including RNAP and the ribosome. These findings highlight chaperones as potential targets for drugs to overcome AMR in mycobacteria, including M. tuberculosis, as well as in other pathogens.

## Abstract (precis)

AMR is a global problem, especially for TB. Here, we show that mycobacterial chaperones support AMR in M. smegmatis, a nonpathogenic model of M. tuberculosis, the causative agent of TB.

## INTRODUCTION

Antimicrobial resistance (AMR) is a threat to public health. Globally, at least 700,000 people per year die from an infection with an organism harboring AMR, and it is estimated that in 30 years, that number will be 10 million (1). In the United States alone, more than 2 million people are infected with a bacterium that is resistant to at least a first-line drug (2). AMR is a concern for those infected with Mycobacterium tuberculosis: about 0.5 million people develop multidrug-resistant (MDR) tuberculosis (TB) annually, and about 9.5% of MDR-TB patients have extensively DR (XDR) TB. Thus, resistance may make the world’s most deadly infectious disease even more deadly. These numbers force an evaluation of the factors that support the fitness of bacteria harboring AMR.

Antimicrobials often inhibit enzymes that catalyze essential cellular processes. This selection pressure leads to emergence of mutants that avoid activating the compound, modify the compound, efflux it, overexpress its target, or change an amino acid in the target that reduces binding of the compound, or it selects for horizontal gene transfer of resistance-encoding genes, etc. The mutations that confer resistance to antimicrobials provide a selective advantage in the presence of the drug but can also lead to protein instability or altered enzyme activity. These mutant enzymes impose an additional stress on the cell, sometimes resulting in reduced fitness of the microorganism *in vitro* and *in vivo* (3) in the absence of the drug. However, microorganisms harboring AMR mutations are *ipso facto* fit enough to survive and divide. In some cases, compensatory mutations ameliorate fitness costs of AMR (4, 5), but there may be a general mechanism in wild-type bacteria that alleviates the fitness cost of AMR.

In 2005, Cowen and Lindquist showed that the eukaryotic protein chaperone Hsp90 plays a role in mediating AMR in diverse fungi (6). When Hsp90 expression was reduced or when its activity was chemically inhibited, resistance to antimicrobials was decreased. This is just one way that chaperones from organisms as diverse as bacteria, yeasts, plants, fruit flies, fish, and humans can buffer genomic diversity (6–13). In *Drosophila*, genetic depletion of Hsp90 or its chemical inhibition led to increased genesis of deformed flies (10). In cave fish, similar intervention results in variations in eye size and even loss of eyes (12). Hsp90 also binds to and helps to fold, stabilize, and activate oncogenic mutant regulators, including v-Src (14, 15), as well as other mutant proteins associated with disease states (16), like FANCA of the Fanconi anemia DNA repair pathway in human cells (13). Chaperones can act globally (stress response) and locally (stabilization of mutant proteins) to maintain cell fitness.

In bacteria, the Hsp90 homolog HtpG plays a less clear role in cell physiology, and only the protein chaperones DnaK (Hsp70 homolog) and GroEL (Hsp60 homolog) have been shown to enable genomic diversity (7–9, 17–21). Overexpression of the *groE* operon in Escherichia coli increased the fitness of strains that had accumulated mutations (7, 17), and deletion of E. coli DnaK slowed resistance evolution to tetracycline (21). When *dinB*, encoding an error-prone DNA polymerase, was expressed in Salmonella enterica serovar Typhimurium, levels of DnaK and GroEL increased in lineages with high mutational burdens (9). These findings suggest that protein chaperones, particularly DnaK and GroEL, maintain the fitness of bacteria with mutations. To our knowledge, however, the role of protein chaperones in supporting the fitness of mycobacteria with mutations that result in AMR has not been explored.

DnaK plays a key role in native protein folding in Mycobacterium smegmatis (22), and protein-folding activity has been reconstituted *in vitro* with M. tuberculosis DnaK and its cochaperones DnaJ1 and DnaJ2 (22, 23). M. smegmatis, a saprophytic mycobacterium, shares mechanisms of resistance similar to those of M. tuberculosis to many anti-TB drugs, so we used it as a model to study AMR in mycobacteria. Herein, we show that the DnaK system (chaperone DnaK and cochaperones DnaJ1 and DnaJ2) in mycobacteria supports AMR. M. smegmatis harboring a mutation in *rpoB*, the target of the first-line TB drug rifampin (RIF), has a dramatic fitness defect when the DnaK cochaperone DnaJ2 is absent. This is also true when the M. smegmatis *rpoB* allele is replaced with a RIF resistance-conferring *rpoB* allele from M. tuberculosis. We show that DnaK associates more with mutant RpoB than with wild-type RpoB, suggesting that the DnaK system may support RIF resistance by directly stabilizing this mutant RNA polymerase (RNAP). Additionally, M. smegmatis harboring a mutation in *rpsL* (streptomycin [SM] target) has a fitness defect when DnaJ2 is absent. Our work confirms a role for the DnaK system in supporting heritable AMR in mycobacteria and provides a rationale for studying the role of protein chaperones in enabling AMR in M. tuberculosis as well as other pathogens.

## RESULTS

### Essential proteins are clients of the DnaK system in mycobacteria.

DnaK plays a key role in native protein folding in M. smegmatis, where the protein is essential (22). To understand why the DnaK system is essential, we identified clients and cochaperones of DnaK by stable isotope labeling with amino acids in cell culture (SILAC) with a M. smegmatis lysine auxotroph expressing 10His-DnaK or 10His-mCherry. These strains also contained a small deletion at the 3′ end of *groEL2* yielding a deletion of the histidine-rich stretch of the GroEL2 C terminus. This deletion prevents copurification of GroEL2 with His-tagged proteins without noticeably impacting the growth of the parent strain (24). In three experiments, SILAC reproducibly yielded 103 potential interactors of DnaK (Table 1; also, see Table S1 in the supplemental material). These proteins were enriched (>1.5 ratio) in 10His-DnaK samples compared to the 10His-mCherry control samples. This protein list includes potential clients and cochaperones and corroborates work done in our lab showing that protein complexes and proteins involved in cell wall biosynthesis are clients for DnaK (22). Unlike similar experiments performed in E. coli (25), M. smegmatis DnaK associated with many essential clients, suggesting that it is a key protein-folding machine in mycobacteria, consistent with its genetic essentiality. Notably, several identified clients are either antimicrobial targets or in pathways inhibited by antimicrobials (Table 1).

**TABLE 1:** Selected proteins identified in SILAC experiments as interacting with M. smegmatis DnaK^*a*^

Protein	General biological process
**FtsZ**	Septum assembly, cytokinesis
**IlvD**, **Ask**, **SerA**, MetH	Amino acid biosynthesis
**MurG**, **GlmU**, UmaA, **AcpM**, **DesA2**	Cell wall (β-lactams, INH, ETA)
**TopA**, TopoN^*b*^	DNA topology (FQ)
**DnaE1**, HspR, HrcA, **MtrA**, MSMEG_6757, 3193^*a*^, 5696	Polymerase/transcription factors/DNA binding
**RpoC**^*c*^, **RpoD**^*c*^, **RpoZ**^*c*^	RNA polymerase (RIF)
**RplA**, -**N**, -**W**, -**Q**, -**K**, -**L**, -**F**, -**X**, -**D**, -I, -**Y**, -**S**, -**C**, -**U**, -**J**, RpsP, -**T**, -**F**, -**I**, -**Q**, -**A**, **Ffh**, PrfC^*b*^	Translation (aminoglycosides, CAP)
**GrpE**, **GroES**, **GroEL2**, GroEL1, Tig	Protein folding
**ClpC1**, **ClpP**	Protein degradation
**PptT**	Phosphopantetheinyl transferase
AhpC	Cell redox homeostasis (INH activation)

a See Table S1 for full list. Essential proteins (M. tuberculosis homologs, as in http://tuberculist.epfl.ch) are in bold and proteins in pathways of antimicrobial action are underlined. The experiment was completed three independent times. INH, isoniazid; ETA, ethionamide; FQ, fluoroquinolone; RIF, rifampin; CAP, capreomycin.

b Not present in M. tuberculosis.

c Identified in two of three SILAC experiments.

10.1128/mBio.00123-21.8 TABLE S1 Full list of proteins identified in SILAC experiments as interacting with M. smegmatis DnaK. Download Table S1, XLSX file, 0.02 MB. Copyright © 2021 Fay et al. 2021 Fay et al. https://creativecommons.org/licenses/by/4.0/ This content is distributed under the terms of the Creative Commons Attribution 4.0 International license.

**FIG 1:**
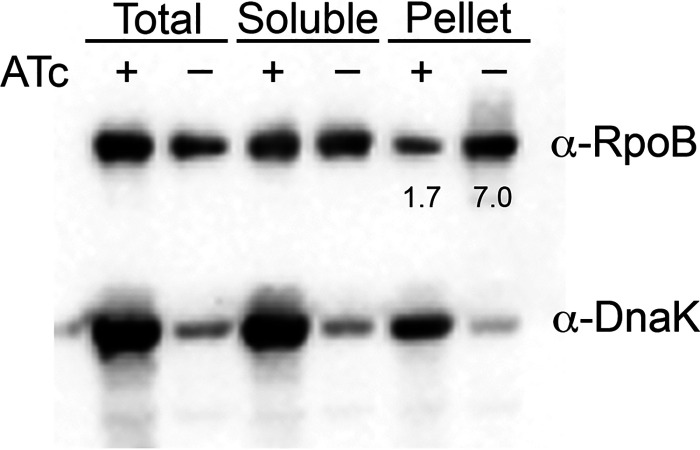
DnaK stabilizes RpoB. Fractionated lysates of M. smegmatis *dnaK* depletion strain (MGM6005) in the presence (+ATC, *dnaK* expression) and absence (−ATC, *dnaK* depletion) of ATC. Lysates probed with anti-RpoB (top) or anti-DnaK (bottom). (Left) Unfractionated lysate (total); (middle) soluble fraction; (right) insoluble fraction (pellet). The numbers under the anti-RpoB pellet bands indicate the ImageJ quantitation as a percentage of the total in this experiment; the average fold increase in RpoB in the pellet (−DnaK/+DnaK) is 3.6. The experiment was performed at least three independent times; a representative image is shown.

If the identified proteins were clients of DnaK, then depletion of DnaK might result in loss of client protein solubility due to destabilization or folding defects. Several components of RNAP were identified in two of three SILAC experiments (Table S1), so we tested whether the DnaK system stabilizes one of the essential proteins in the RNAP complex, RpoB. We depleted DnaK using our anhydrotetracycline (ATC)-regulated *dnaK* depletion strain of M. smegmatis (22), fractionated the lysates from DnaK-depleted and -replete cells, and analyzed RpoB by immunoblotting. RpoB accumulated in the pellet/insoluble fraction upon DnaK depletion (Fig. 1), confirming that this essential protein required the mycobacterial DnaK system for stability.

### The DnaK system buffers the fitness defect of a RIF resistance-conferring AMR mutation in M. smegmatis.

**FIG 2:**
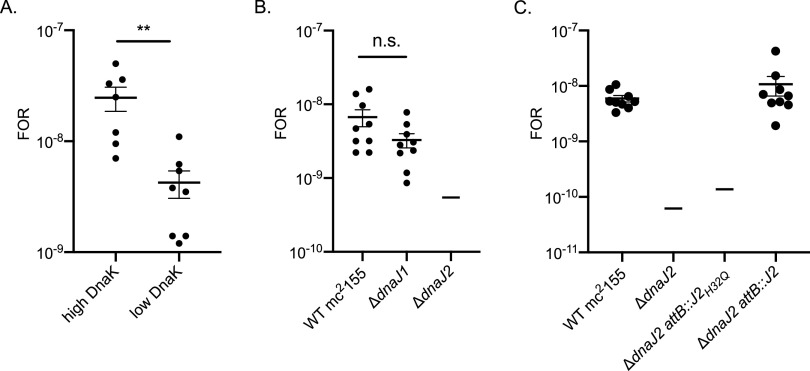
The DnaK chaperone system supports resistance to RIF in M. smegmatis. (A) Frequency of resistance (FOR) to RIF for the DnaK depletion strain. High DnaK, cells grown in 25 ng/ml ATC; low DnaK, cells grown in 0.78 ng/ml ATC (see Fig. S1). (B) FOR to RIF for wild-type M. smegmatis and M. smegmatis lacking DnaJ1 or DnaJ2. The Δ*dnaJ2* strain gave no colonies on RIF at 200 μg/ml; the line indicates the LOD at 5.4 × 10^−10^. (C) FOR to RIF for wild-type M. smegmatis, M. smegmatis lacking DnaJ2, and M. smegmatis lacking DnaJ2 expressing *dnaJ2*_H32Q_ or *dnaJ2*. Δ*dnaJ2* and Δ*dnaJ2* strains expressing *dnaJ2*_H32Q_ gave no colonies on RIF at 200 μg/ml; lines denote LOD of 6.2 × 10^−11^ (Δ*dnaJ2*) and 1.4 × 10^−10^ (Δ*dnaJ2 attB*::*J2*_H32Q_). Data are means and standard errors of the means (SEM) from three independent experiments. Statistical significance was calculated by using Student's *t* test (**, *P < *0.01; n.s., not significant).

In the DnaK SILAC experiment, three subunits of RNAP associated with DnaK, as well as phosphopantetheinyl transferase, PptT, suggesting that DnaK may play a role in promoting folding and/or stability of these proteins. For these reasons, we tested the frequency of resistance (FOR) of our M. smegmatis *dnaK* depletion strain (22) to RIF, whose target is RpoB of RNAP, and to 8918, a compound that was identified in a high-throughput screen and found to target PptT (26). We hypothesized that if DnaK is required for folding and/or maintaining stability of the wild-type proteins, then reduced DnaK levels may destabilize mutant proteins further, put additional stress on the cell, and therefore affect the FOR. The FOR to RIF correlated with the levels of DnaK: elevated DnaK levels led to high FOR; reducing DnaK levels resulted in ∼6-fold-lower FOR (Fig. 2A). This trend was not observed with the compound 8918 (2 × 10^−7^ FOR for both high and low DnaK), perhaps because 90% of resistance-conferring mutations to this compound were not in PptT but in an adjacent gene that regulates the same pathway (26). The product of the adjacent gene, PptH, was not identified as a DnaK-interacting protein by SILAC. As opposed to FOR, the level of DnaK did not alter M. smegmatis’s susceptibility to RIF or 8918: the MIC and the number of CFU at 2× MIC were the same for each strain (Fig. S1; Table S2). Thus, loss of DnaK specifically affects the evolution of resistance, not the MIC for mycobacteria to the antimicrobials.

10.1128/mBio.00123-21.1 FIG S1 DnaK level does not affect the efficiency of killing of M. smegmatis by RIF or 8918. Download FIG S1, PDF file, 0.07 MB. Copyright © 2021 Fay et al. 2021 Fay et al. https://creativecommons.org/licenses/by/4.0/ This content is distributed under the terms of the Creative Commons Attribution 4.0 International license.

10.1128/mBio.00123-21.9 TABLE S2 List of strains, plasmids, oligonucleotides, competition experiments, and relevant MICs. Download Table S2, XLSX file, 0.02 MB. Copyright © 2021 Fay et al. 2021 Fay et al. https://creativecommons.org/licenses/by/4.0/ This content is distributed under the terms of the Creative Commons Attribution 4.0 International license.

DnaJ1 and DnaJ2 are cochaperones for the DnaK system in mycobacteria (22); they present client proteins to DnaK for refolding. Individually, they are not essential for M. smegmatis growth, but they are collectively essential for growth (22). We hypothesized that one or both of the cochaperones aid DnaK in supporting RIF resistance in M. smegmatis. To identify which DnaK cochaperone is responsible for the observed difference in FOR to RIF, we tested the FOR of the Δ*dnaJ1* and Δ*dnaJ2* backgrounds of M. smegmatis to RIF. The FOR to RIF was similar for wild-type M. smegmatis and the Δ*dnaJ1* strain (Fig. 2B); however, we did not detect any mutants on RIF plates for the Δ*dnaJ2* strain (limit of detection [LOD] = 5.4 × 10^−10^ in Fig. 2B and LOD = 6.2 × 10^−11^ in Fig. 2C). The FOR to RIF was restored to wild-type levels upon complementation with *dnaJ2* integrated at *attB* (Fig. 2C). Notably, the MICs of RIF for these strains are similar to those for the wild type (Table S2).

To confirm that DnaJ2 cochaperone activity is required for the formation of RIF resistance mutants, we complemented Δ*dnaJ2* with *dnaJ2*_H32Q_, which we have shown does not support DnaJ essential function in M. smegmatis and failed to stimulate DnaK ATPase activity *in vitro* (23). M. smegmatis expressing DnaJ2_H32Q_ in the Δ*dnaJ2* background did not yield any colonies on RIF plates (LOD = 1.4 × 10^−10^) (Fig. 2C), suggesting that DnaJ2 activity is required for the survival of RIF-resistant cells in M. smegmatis.

**FIG 3:**
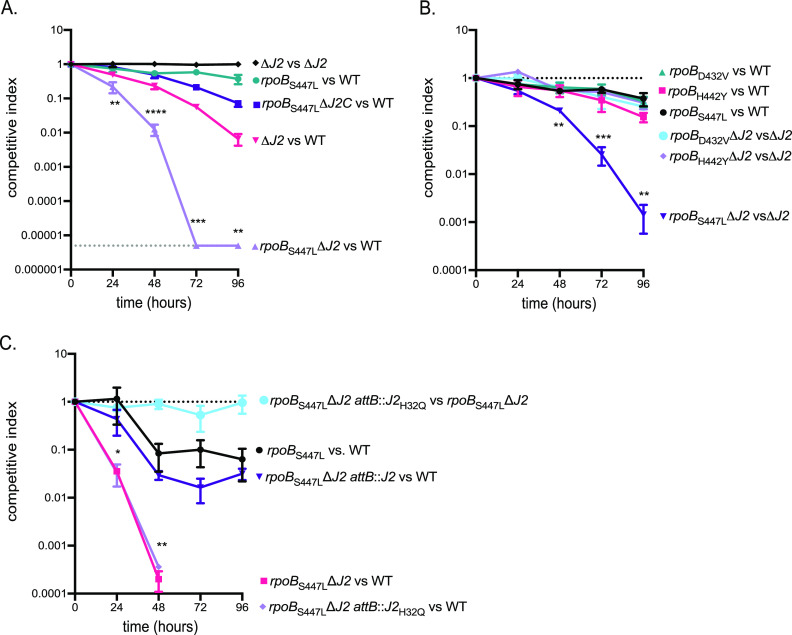
Competition experiments reveal a fitness defect for M. smegmatis *rpoB*_S447L_ lacking active DnaJ2. (A) Competitive growth indexes of RIF resistance strains in wild-type and Δ*dnaJ2* backgrounds. Note that the *rpoB*_S447L_Δ*dnaJ2* strain did not have detectable CFU after 48 h when competed with the wild type. MGM6544 versus MGM6545 (black), MGM6510 versus MGM6133 (teal), MGM6503 versus MGM6543 (purple), MGM6545 versus MGM6133 (pink), MGM6502 versus MGM6543 (lavender). (B) Competitive growth indexes of *rpoB*_S447L_ and Δ*dnaJ2* strains. MGM6547 versus MGM6133 (black), MGM6546 versus MGM6133 (pink), MGM6510 versus MGM6133 (black), MGM6541 versus MGM6545 (light blue), MGM6537 versus MGM6545 (lavender), MGM6502 versus MGM6545 (purple). (C) Competitive growth indexes of the *rpoB*_S447L_ strain expressing DnaJ2 or inactive DnaJ2_H32Q_. MGM6969 versus MGM6502 (light blue), MGM6510 versus MGM6133 (black), MGM6968 versus MGM6133 (purple), MGM6566 versus MGM6133 (pink), MGM6969 versus MGM6133 (lavender). A competitive index of 1 indicates equal fitness. The graph represents an independent experiment performed in triplicate. Each experiment was performed three times (except that for panel C, which was performed two times) with similar results. Values are means and standard deviations (SD). Statistical significances were calculated using Student's *t* test comparing MGM6502 versus MGM6543 (lavender) to MGM6510 versus MGM6133 (teal) (A); MGM6502 versus MGM6545 (purple) to MGM6547 versus MGM6133 (black) (B); and MGM6969 versus MGM6133 (lavender) to MGM6968 versus MGM6133 (purple) (C) (*, *P < *0.05; **, *P < *0.01; ***, *P < *0.001; ****, *P < *0.0001).

*In vitro*, many RNAP mutations result in resistance to RIF (27, 28), yet only a few are found in RIF-resistant clinical isolates of M. tuberculosis. To directly test the role of the DnaK system in buffering the fitness cost of specific point mutations, we generated mutant *rpoB* alleles encoding D432V, H442Y, and S447L mutations (corresponding to the most common mutations in RIF-resistant clinical isolates in M. tuberculosis [29–31]) in the Δ*dnaJ1* and Δ*dnaJ2* backgrounds of M. smegmatis (23) by recombineering. We used the Δ*dnaJ* strains as surrogates for the DnaK depletion strain to avoid the selection of deregulated mutants that arise from serial passaging of DnaK depletion cultures. We used a coculture competition assay to test the relative fitness of the strains. In competition experiments, the *rpoB*_S447L_ strain had a slight fitness defect when competed with the wild-type parent strain (Fig. 3A, *rpoB*_S447L_ versus wild type [WT]). However, loss of *dnaJ2* in the *rpoB*_S447L_ strain resulted in a >5-log_10_ defect when it was competed with the wild type after 72 h (Fig. 3A, *rpoB*_S447L_Δ*J2* versus WT), a defect that was ∼4 log_10_ more severe than the fitness defect of Δ*dnaJ2* (Δ*J2* versus WT). The fitness defect of the *rpoB*_S447L_Δ*dnaJ2* strain was restored upon complementation with *dnaJ2* integrated at *attB* (Fig. 3A, *rpoB*_S447L_Δ*J2C* versus WT). This defect in fitness was not observed for the *rpoB*_S447L_ strain in the Δ*dnaJ1* background (Fig. S2). When competed with the Δ*dnaJ2* mutant, the *rpoB*_S447L_ strain had an ∼3-log_10_ fitness defect in the absence of DnaJ2 (*rpoB*_S447L_Δ*J2* versus Δ*J2*), whereas D432V and H442Y did not (Fig. 3B). Similar to the *rpoB*_S447L_ strain, the *rpoB*_D432V_ and *rpoB*_H442Y_ strains had a slight fitness defect when competed with the wild type (32–34) (Fig. 3B, *rpoB*_X_ versus WT). The expression of the mutant alleles was not altered in the chaperone deletion background (Fig. S3). Notably, the *rpoB*_S450L_ mutation (*rpoB*_S447L_ in M. smegmatis) is found in ∼50% of RIF-resistant M. tuberculosis clinical isolates (35–37).

10.1128/mBio.00123-21.2 FIG S2 Competition experiments reveal no fitness defect for M. smegmatis *rpoB*_S447L_ in the absence of DnaJ1. Download FIG S2, PDF file, 0.04 MB. Copyright © 2021 Fay et al. 2021 Fay et al. https://creativecommons.org/licenses/by/4.0/ This content is distributed under the terms of the Creative Commons Attribution 4.0 International license.

10.1128/mBio.00123-21.3 FIG S3 RpoB levels in M. smegmatis strains. Download FIG S3, PDF file, 0.4 MB. Copyright © 2021 Fay et al. 2021 Fay et al. https://creativecommons.org/licenses/by/4.0/ This content is distributed under the terms of the Creative Commons Attribution 4.0 International license.

To confirm that DnaJ2 cochaperone activity is required for the fitness of the *rpoB*_S447L_ strain, we complemented *rpoB*_S447L_Δ*J2* with *dnaJ2*_H32Q_. In contrast to wild-type DnaJ2, expression of DnaJ2_H32Q_ did not restore fitness to the *rpoB*_S447L_Δ*J2* strain (Fig. 3C), suggesting that DnaJ2 activity is required for the fitness of this mutant.

### The DnaK system buffers the fitness cost of M. tuberculosis RNAP RpoB_S450L_ AMR mutation in M. smegmatis.

**FIG 4:**
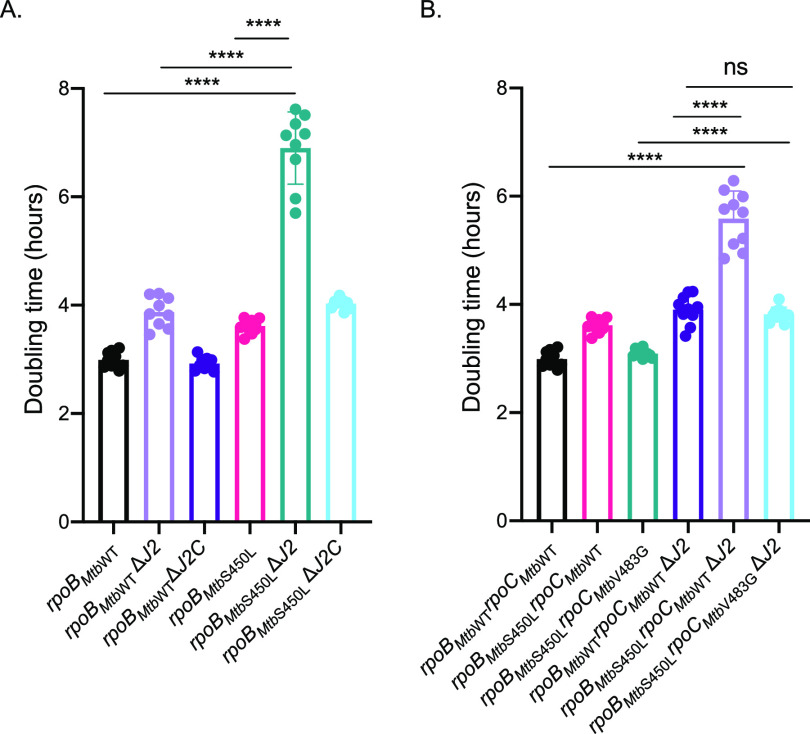
Doubling time measurements reveal a fitness defect for M. smegmatis *rpoB_Mtb_*_S450L_ in the absence of DnaJ2. (A) Doubling times for *rpoB_Mtb_*_WT_ and *rpoB_Mtb_*_S450L_ in wild-type, Δ*dnaJ2*, and Δ*dnaJ2* complemented backgrounds. TS106 (black), MGM7068 (lavender), MGM7069 (purple), TS108 (pink), MGM7072 (teal), MGM7073 (light blue). (B) Doubling times for M. smegmatis strains containing RIF resistance mutation *rpoB_Mtb_*_S450L_ and its compensatory mutation *rpoC_Mtb_*_V483G_ in wild-type and Δ*dnaJ2* backgrounds. TS106 (black), TS108 (pink), TS113 (teal), MGM7028 (purple), MGM7031 (lavender), MGM7029 (light blue). The experiment was performed three times in triplicate for each strain. Values are means and SD. Statistical significances were calculated using the Wilcoxon rank-sum test (Mann-Whitney *U* test) (****, *P < *0.0001; ns, not significant).

Our attempt to test the role of the DnaK system in supporting AMR in M. tuberculosis was hindered by the formation of suppressor mutations in M. tuberculosis strains that lack the cochaperone DnaJ2 (our unpublished observations). Because of this limitation, we obtained the published recombinant M. smegmatis strain that has a deletion of its own *rpoB* and *rpoC* alleles and expresses the M. tuberculosis *rpoB*_WT_ *or rpoB*_S450L_ (S447L in M. smegmatis) and *rpoC* alleles at the *attB* site (referred to here as *rpoB_Mtb_*_WT_ or *rpoB_Mtb_*_S450L_) (33). We used the M. smegmatis *rpoB_Mtb_* strains to assess whether the DnaK system supports the M. tuberculosis *rpoB_Mtb_*_S450L_ allele in M. smegmatis by comparing growth rates. We used growth rates to measure fitness here because addition of selective markers in these strains resulted in a slow growth phenotype, and literature suggests that growth rate comparison can be used to substitute for competitive fitness assays (38). As observed previously (33), *rpoB_Mtb_*_S450L_ had a slight fitness defect, as evidenced by the apparent doubling time, compared to *rpoB_Mtb_*_WT_ (Fig. 4A). Deletion of *dnaJ2* in the M. smegmatis *rpoB_Mtb_*_S450L_ strain (*rpoB_Mtb_*_S450L_Δ*J2*) resulted in a doubling time of 6.89 ± 0.67 h, which was a 78% percent increase compared to *rpoB_Mtb_*_WT_Δ*dnaJ2*; this was more than the growth defect of Δ*dnaJ2* (29% increase compared to *rpoB_Mtb_*_WT_) and *rpoB_Mtb_*_S450L_ (20% increase compared to *rpoB_Mtb_*_WT_) strains combined (29 + 20 = 49%). The fitness defect of the *rpoB_Mtb_*_S447L_Δ*dnaJ2* strain was lost upon complementation with *dnaJ2* (*rpoB_Mtb_*_WT_Δ*J2C*) but not with the empty vector control pMV261kan. The levels of RpoB remained similar between WT and S450L as well as in the chaperone deletion background (Fig. S4). These data suggest that the fitness defect imparted by the RIF resistance-conferring *rpoB*_S447L_ allele in M. smegmatis is also imparted by the *rpoB_Mtb_*_S450L_ allele from M. tuberculosis and that the mycobacterial DnaK system of chaperones alleviates this defect.

10.1128/mBio.00123-21.4 FIG S4 RpoB levels in strains carrying M. tuberculosis *rpoBC* alleles. Download FIG S4, PDF file, 0.7 MB. Copyright © 2021 Fay et al. 2021 Fay et al. https://creativecommons.org/licenses/by/4.0/ This content is distributed under the terms of the Creative Commons Attribution 4.0 International license.

### A compensatory mutation still compensates for the fitness defect of *rpoB_Mtb_*_S450L_ in the absence of the DnaK system.

Compensatory mutations in the RNAP subunits *rpoC* and *rpoA* have been shown to ameliorate fitness costs of RIF-resistant M. tuberculosis *in vitro* and in the clinic (4, 5, 33, 39, 40). To test whether compensatory mutations alleviate the fitness defect of M. smegmatis harboring *rpoB_Mtb_*_S450L_ in the Δ*dnaJ2* background, we used the recombinant M. smegmatis strain described above (M. smegmatis *rpoB_Mtb_*_S450L_) that carries the compensatory *rpoC* allele *rpoC_Mtb_*_V483G_ at the *attB* site (33). The V483G substitution and many of the substitutions in RpoC that compensate for the RpoB S450L mutation are in the interface of the β and β′ subunits of RNAP. It was suggested that these compensatory mutations could restore structural interactions in subunits of RNAP (40). Addition of the *rpoC_Mtb_*_V483G_ compensatory mutation to M. smegmatis *rpoB_Mtb_*_S450L_Δ*J2* restored the doubling time of M. smegmatis back to levels of the Δ*dnaJ2* strain (Fig. 4B, *rpoB_Mtb_*_S450L_*rpoC_Mtb_*_V483G_Δ*J2*). These data suggest that the amino acid substitution V483G in RpoC still compensates for the fitness defect imparted by the S450L RpoB RIF resistance-conferring substitution in the absence of DnaJ2. Notably, RpoC V483G did not compensate for the fitness defect imparted by the *dnaJ2* deletion alone.

### Enhanced association of RpoB_S447L_ with DnaK.

**FIG 5:**
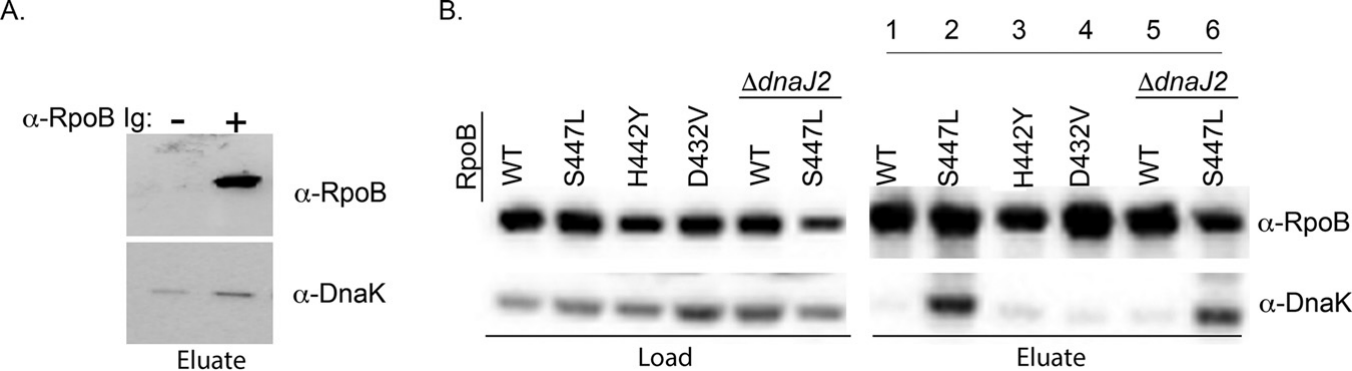
DnaK associates with RpoB in M. smegmatis. (A) Immunoprecipitation of RpoB from M. smegmatis wild-type MC^2^155. Anti-GFP (mouse, monoclonal) was used in lysate as a nonspecific Ig control. (B) Immunoprecipitation of RpoB from M. smegmatis *rpoB*_WT_ (lane 1, MC^2^155), *rpoB*_S447L_ (lane 2, MGM6465), *rpoB*_H447Y_ (lane 3, MGM6466), *rpoB*_D432V_ (lane 4, MGM6467), *rpoB*_WT_Δ*dnaJ2* (lane 5, MGM6301), and *rpoB*_S447L_Δ*dnaJ2* (lane 6, MGM6481). Membranes were probed as indicated. Pulldown experiments were performed three independent times; a representative image is shown.

Our findings suggest that the DnaK system supports the fitness of the RIF resistance-conferring *rpoB*_S447L_ mutation and the *rpoB_Mtb_*_S450L_ mutation in M. smegmatis. Because several subunits of RNAP were identified in the DnaK SILAC experiments (Table 1), we hypothesized that the DnaK system directly binds to and stabilizes RpoB. To test this idea, we immunoprecipitated M. smegmatis RpoB and tested for coprecipitation of DnaK by immunoblotting. DnaK associated with RpoB_WT_ compared with the Ig control, confirming the interaction (Fig. 5A). We performed the same experiment in cells harboring the RIF resistance-conferring *rpoB*_D432V_, *rpoB*_H442Y_, or *rpoB*_S447L_ mutation. RpoB_D432V_ and RpoB_H442Y_ associated with similar levels of DnaK as the WT, whereas RpoB_S447L_ associated with more DnaK than RpoB_WT_ (Fig. 5B).

The increased association of DnaK with RpoB_S447L_ corroborates our fitness data for the *rpoB*_S447L_ strain; it is also consistent with recently published crystal structures of M. smegmatis RNAP with RpoB_S447L_ that demonstrate considerable disorder compared to the structures of M. smegmatis RNAP with RpoB_WT_ (41). We hypothesized that in the absence of DnaJ2, the DnaK system would no longer associate with RpoB_S447L_, resulting in the observed fitness defect of this strain (Fig. 3). To test the association between RpoB_S447L_ and DnaK in the absence of DnaJ2, we immunoprecipitated M. smegmatis RpoB and blotted for DnaK in the Δ*dnaJ2* strain. Even in cells lacking the cochaperone DnaJ2, RpoB_S447L_ still associated with DnaK (Fig. 5B). Furthermore, more DnaK did not associate with RpoB*_Mtb_*_S450L_ than with RpoB*_Mtb_*_WT_ (Fig. S5). These data suggest that the fitness defect observed for *rpoB*_S447L_Δ*dnaJ2* and for *rpoB_Mtb_*_S450L_Δ*dnaJ2* is not due to the inability of RpoB_S447L_ or RpoB*_Mtb_*_S450L_ to associate with DnaK.

10.1128/mBio.00123-21.5 FIG S5 DnaK does not associate more with RpoB*_Mtb_*_S450L_ than RpoB*_Mtb_*_WT_ in M. smegmatis. Download FIG S5, PDF file, 1.4 MB. Copyright © 2021 Fay et al. 2021 Fay et al. https://creativecommons.org/licenses/by/4.0/ This content is distributed under the terms of the Creative Commons Attribution 4.0 International license.

### DnaK system buffers another AMR mutation in M. smegmatis.

**FIG 6:**
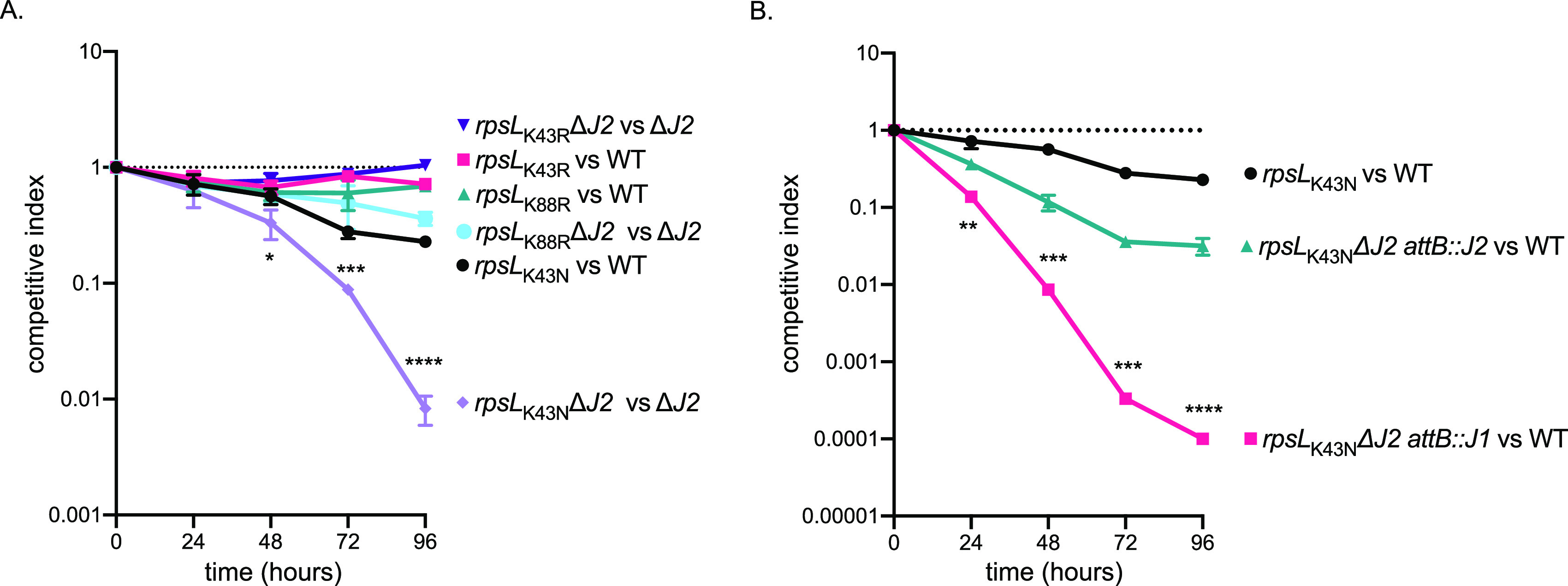
Competition experiments reveal a fitness defect for M. smegmatis *rpsL*_K43N_ in the absence of DnaJ2. (A) Competitive growth indexes of streptomycin resistance strains in wild-type and Δ*dnaJ2* backgrounds. MGM6581 versus MGM6580 (purple), MGM6577 versus MGM6575 (pink), MGM6578 versus MGM6575 (teal), MGM6583 versus MGM6580 (light blue), MGM6576 versus MGM6575 (black), MGM6582 versus MGM6580 (lavender). (B) Complementation of the Δ*dnaJ2* allele with *dnaJ2* or *dnaJ1*. MGM6576 versus MGM6575 (black), MGM6615 versus MGM6575 (teal), MGM6614 versus MGM6575 (pink). A competitive index of 1 indicates equal fitness. The graph represents independent experiment performed in triplicate. The experiment was performed twice. Values are means and SD. Statistical significances were calculated using Student's *t* test comparing MGM6576 versus MGM6575 (black) to MGM6582 versus MGM6580 (lavender) (A) and MGM6576 versus MGM6575 (black) to MGM6614 versus MGM6575 (pink) (B) (*, *P < *0.05; **, *P < *0.01; ***, *P < *0.001; ****, *P < *0.0001).

To extend our analysis of the DnaK system in mediating AMR in mycobacteria to other drug targets, we looked at *rpsL* mutations that confer high-level resistance to the broad-spectrum aminoglycoside SM, which is a second-line TB drug that inhibits protein synthesis by targeting the ribosome (42). We generated mutant *rpsL* alleles encoding K43N, K43R, and K88R mutations in the Δ*dnaJ1* and Δ*dnaJ2* backgrounds of M. smegmatis and asked whether the mutations impair the ability of these strains to compete with wild-type strains bearing the same *rpsL* mutations in standard 7H9 medium. These *rpsL* mutations are found in clinical isolates of SM-resistant M. tuberculosis. M. smegmatis Δ*dnaJ2* with the *rpsL*_K43N_ SM resistance-conferring mutation had an ∼2 log_10_ fitness defect when competed with Δ*dnaJ2* (Fig. 6A). This defect was complemented with a plasmid harboring *dnaJ2*, but not *dnaJ1* (Fig. 6B), and was not observed in the Δ*dnaJ1* mutant (Fig. S6). The other SM resistance-conferring mutations tested, *rpsL*_K43R_ and *rpsL*_K88R_, did not show a fitness defect in cells lacking DnaJ2 or DnaJ1.

10.1128/mBio.00123-21.6 FIG S6 Competition experiments reveal no fitness defect for streptomycin resistance alleles in M. smegmatis in the absence of DnaJ1. Download FIG S6, PDF file, 0.04 MB. Copyright © 2021 Fay et al. 2021 Fay et al. https://creativecommons.org/licenses/by/4.0/ This content is distributed under the terms of the Creative Commons Attribution 4.0 International license.

Notably, M. smegmatis lacking DnaJ2 with the fluoroquinolone resistance-conferring mutations *gyrA*_S91P_, *gyrA*_D94G_, or *gyr*A**_A90V_ did not exhibit a fitness defect when competed with the Δ*dnaJ2* or Δ*dnaJ1* strain (Fig. S7A). In addition, there was no fitness defect of the isoniazid resistance-conferring mutation *inhA*_S94A_ in the wild-type, Δ*dnaJ1*, or Δ*dnaJ2* background (Fig. S7B).

10.1128/mBio.00123-21.7 FIG S7 Competition experiments reveal no fitness defect for fluoroquinolone resistance alleles or an isoniazid resistance allele in M. smegmatis lacking DnaJ1 or DnaJ2. Download FIG S7, PDF file, 0.10 MB. Copyright © 2021 Fay et al. 2021 Fay et al. https://creativecommons.org/licenses/by/4.0/ This content is distributed under the terms of the Creative Commons Attribution 4.0 International license.

## DISCUSSION

We found that mycobacterial DnaK associates with many essential proteins, including the targets of antimicrobials or proteins in pathways that are targeted by antimicrobials. DnaK also associates with many putative transcription factors. These findings explain the essentiality of the DnaK system in mycobacteria and encouraged us to pursue the hypothesis that the mycobacterial DnaK system could support the survival of cells containing mutant alleles of essential cellular proteins that are the targets of antimicrobials. We hypothesized that DnaK could support AMR in mycobacteria by either directly binding to proteins that contain resistance-conferring amino acid substitutions or by acting globally to stabilize the stress response to these substitutions.

Resistance to RIF is increasing. In 2018, the WHO reported about 0.5 million new cases of RIF-resistant TB. We found that the DnaK chaperone system plays an important role in supporting resistance to RIF in M. smegmatis: RIF resistance was less frequent in cells depleted of DnaK, and we were unable to isolate any RIF-resistant mutants in bacteria lacking the DnaK cochaperone DnaJ2. The most prevalent resistance mutation observed in the clinic for patients with RIF-resistant TB is the *rpoB* S450L mutation (S447L in M. smegmatis). Our data indicate that the fitness of mycobacteria with the *rpoB*_S447L_ allele (M. smegmatis) or the *rpoB*_MtbS450L_ allele (M. tuberculosis) decreases dramatically when cells lack DnaJ2. The fitness defect is specific for this RIF resistance allele and specific for the cochaperone DnaJ2. The presence of DnaJ2 does not alter the ability of a compensatory mutation (RpoC_V438G_) to compensate for the fitness defect of RpoB_S450L_; however, mycobacteria that harbor the RpoB_S450L_ and RpoC_V438G_ mutations still require DnaJ2 for optimal fitness. In addition, we found that DnaK associates with and stabilizes RpoB. Although RpoB, RpoD and RpoZ did not reach the cutoff to be classified as a hit in the DnaK SILAC experiments, RpoC was identified as a hit in two of three experiments (Table 1; Table S1). We found that DnaK associates more with RpoB_S447L_ than RpoB_WT_. Intriguingly, structural analysis of M. smegmatis RNAP harboring RpoB_S447L_ in the absence of DNA reveals disorder in the β-subunit loop (41), supporting the need for the DnaK system in the fitness of cells with this mutation. E. coli RNAP was shown to associate with DnaK (43), and DnaK protects E. coli RNAP from heat denaturation and reactivates heat-denatured RNAP *in vitro* (44), suggesting that the association between chaperone and RNAP (wild-type and AMR-conferring alleles) may be extended to additional microbes. The enhanced association between RpoB_S447L_ and DnaK was specific for RpoB_S447L_; it was not observed with RpoB*_Mtb_*_S450L_. It was also observed in the absence of cochaperone DnaJ2. Therefore, it does not provide an explanation for the fitness defect of strains harboring the S447L or MtbS450L mutations in the absence of DnaJ2.

Work by Stefan et al. found that at the molecular level, RNAP containing RpoB_S450L_ had decreased open complex stability and a decreased elongation rate and was more efficient at intrinsic termination than complexes with RpoB_WT_ (45). The RpoC_V438G_ mutation could compensate for the open complex instability as well as the decreased elongation rate; it could not fully compensate for the increased efficiency of intrinsic termination. It is possible that DnaJ2 helps to stabilize the termination complex of RNAP containing RpoB_S450L_. It is also possible that the DnaK system in M. smegmatis works more broadly and is able to mitigate downstream stress imparted by the RpoB_S447L_ or RpoB_S450L_ mutation. M. tuberculosis harboring the *rpoB*_S450L_ (M. smegmatis *rpoB*_S447L_) allele has transcriptional and metabolomic signatures indicative of altered phthiocerol dimycocerosates (PDIMs) and various lipids (46, 47). Previous data suggest that higher-molecular-weight proteins and protein complexes are likely substrates for M. smegmatis DnaK (22); it is plausible that the *rpoB*_S447L_ or *rpoB_Mtb_*_S450L_ allele alters the transcriptional and proteomic landscape of mycobacteria and results in an enhanced dependence on the DnaK chaperone system. Future work will address this possibility.

In addition to the fitness defect observed with cells expressing RpoB_S447L_ in the absence of DnaJ2, cells expressing the SM resistance-conferring RpsL K43N mutation also show a fitness defect in the absence of DnaJ2. Several subunits of the ribosome were identified in our experiments as DnaK-interacting proteins, so it is possible that the ribosome is a substrate for folding or assembly by the DnaK system; E. coli DnaK has been shown to facilitate ribosome biogenesis (48–50). However, we did not observe alterations in the level or stability of RpsI, a component of the ribosome, upon DnaK depletion in M. smegmatis (data not shown). Ribosomes with the RpsL K43N mutation display enhanced translational fidelity (51). It is possible that alterations in the ribosome capacity or activity in this mutant put an increased dependence on the DnaK system, and this results in a fitness defect in the absence of DnaJ2.

We did not observe fitness defects upon chaperone depletion for three clinically relevant fluoroquinolone resistance mutations or for the *inhA* mutation that confers resistance to isoniazid. Neither gyrase nor InhA was identified as a DnaK client in the SILAC experiment; it is possible that these mutations do not alter the cellular physiology enough to require buffering, or other chaperones could be responsible for buffering the effects of these mutations.

The effects that we observed are specific to the cochaperone DnaJ2; we did not observe fitness defects in any of the mutations tested with DnaJ1. This could be because we have not tested enough mutants, or it could suggest that DnaJ2 plays a bigger role in mycobacterial physiology than DnaJ1. DnaJ proteins control the ATPase activity of DnaK and assist in the delivery of substrate proteins for refolding by DnaK. Inactivating mutations of either DnaJ1 or DnaJ2 results in a slight growth defect in M. smegmatis; however, *in vitro* studies with purified protein (23) and overexpression studies in M. tuberculosis (52) revealed differences between the two J protein activities. Our findings are consistent with these previous studies. Future work will focus on substrate differences between DnaJ1 and DnaJ2 in mycobacteria.

Protein chaperones aid in protein folding, maintenance of protein integrity, and cellular stress response; in doing so, they have the unique ability to directly stabilize resistance-conferring amino acid substitutions in drug targets and counter the stress imparted by these substitutions. Numerous studies report a role for protein chaperones, in particular, Hsp90, in supporting amino acid substitutions in diverse organisms and cell types (6–13, 21). In the pathogenic fungus Candida albicans, Hsp90 was shown to play a role in AMR to the azoles (6) and the echinocandins (53); in E. coli, DnaK was shown to play a role in the evolution of resistance to tetracycline (21). Herein, we show that the DnaK protein chaperone system in mycobacteria supports the fitness of strains harboring clinically relevant resistance-conferring amino acid substitutions in RpoB and the ribosome, the targets of first-line (RIF) and second-line (SM) TB drugs. In general, lower *in vitro* fitness correlates with lower fitness in the clinic for M. tuberculosis (54), which suggests that the cost of resistance determines the spread of DR TB. To our knowledge, this is the first report on the role of protein chaperones in supporting AMR in mycobacteria. Although technical limitations prevented us from testing the role of the DnaK system in supporting AMR in M. tuberculosis, we did observe a fitness defect in M. smegmatis harboring the M. tuberculosis RIF resistance-conferring *rpoB* allele in the absence of the DnaK system. This study supports future studies aimed at inhibiting the DnaK system to sensitize or prevent DR in M. tuberculosis, and perhaps other bacteria (55). Interestingly, M. tuberculosis encodes an Hsp90 homolog HtpG (Rv2299c) (56). Future studies aim to determine the role of the DnaK system and HtpG in supporting AMR in this pathogen. Finally, given the widespread role of chaperones in enabling genomic diversity (6–13, 21), we anticipate that our findings can be extended to other microbes.

## MATERIALS AND METHODS

### Growth conditions.

M. smegmatis strains were cultured in LB with 0.5% glycerol, 0.5% dextrose, and 0.05% Tween 80 (LB_smeg_) or Difco Middlebrook 7H9 supplemented with 0.2% glycerol, 10% ADS (0.5% albumin, 0.085% NaCl, 0.2% dextrose) and 0.05% Tween 80 (7H9). Difco Middlebrook 7H10 or 7H11 agar plates were supplemented with 0.2% glycerol and 10% (wt/vol) Middlebrook oleic acid-albumin-dextrose-catalase (OADC).

### Strain construction (AMR alleles).

Strains used in this study are listed in Table S2. The M. smegmatis DnaK depletion strain and the DnaJ1 and DnaJ2 knockout strains are described in reference 22. For RIF resistance-conferring *rpoB* alleles and isoniazid (INH) resistance-conferring *inhA* alleles, strains were made by oligonucleotide recombineering and selection on RIF (100 μg/ml) or INH (10 μg/ml). Mutations were confirmed by sequencing *rpoB* or *inhA*, and confirmed strains were subsequently passaged on sucrose to select for loss of the recombineering plasmid, pAJF519, a derivative of pRGM18 (57) with the hygromycin resistance cassette removed. For SM resistance-conferring *rpsL* alleles, spontaneous mutants were selected on SM (20 μg/ml) plates. Mutations were identified by sequencing *rpsL*. Quinolone resistance-conferring *gyrA* alleles were made in two ways. In wild-type and Δ*dnaJ2* backgrounds, spontaneous mutants were selected on ofloxacin (0.5 μg/ml) plates. In wild-type and Δ*dnaJ1* backgrounds, the endogenous *gyrAB* locus deletion was made by homologous recombination and double-negative selection (58) in a merodiploid containing a second copy of *gyrAB* at *attB*. Quinolone resistance-conferring *gyrA* alleles were then swapped for the WT copy at *attB*.

### SILAC.

M. smegmatis was grown for 24 h in 7H9 to an optical density at 600 nm (OD_600_) of 0.5 with 40 μg/ml lysine (light or heavy) and then diluted to an OD_600_ of 0.007 in the same media. Cultures were cooled on ice and harvested by centrifugation. The cell pellets were washed with buffer A (25 mM Tris, 200 mM NaCl; pH 8.0) and suspended in buffer A with 200 U apyrase. The samples were lysed with a French press (passing twice at 16,000 lb/in^2^); 0.1% Triton X-100 was added and mixed for 15 min. The debris was pelleted (3,700 × *g*, 20 min) and the supernatant was used as the starting material for SILAC. The lysate was added to HisPur slurry and incubated for 1 h with mixing. Unbound lysate was allowed to flow through, and the resin was washed in buffer A with 0.1% Triton X-100. Bound proteins were eluted in buffer A containing 250 mM imidazole. The sample was diluted, bound to fresh resin again, washed with buffer A, and eluted with buffer A containing 250 mM imidazole. Equal volumes of eluate from heavy and light samples were combined, concentrated, and submitted to the MSKCC Proteomics Core for analysis.

Proteins were separated by SDS-PAGE and stained with Simply Blue (Life Technologies), and 10 gel sections were excised with *in situ* trypsin digestion of polypeptides in each gel slice performed as described elsewhere (59). The tryptic peptides were desalted using stage tips (Thermo Scientific) following the manufacturer’s instructions. The purified peptides were diluted to 0.1% formic acid, and each gel section was analyzed separately by microcapillary liquid chromatography (LC) with tandem mass spectrometry (MS) by using the NanoAcquity system (Waters) with a 100-μm inner diameter, 10-cm-long C_18_ column (1.7 μm particle size; BEH130; Waters) configured with a 180-μm by 2-cm trap column coupled to an Orbitrap Elite or QE Plus mass spectrometer (Thermo Fisher Scientific). Peptides were eluted with a 0-to-50% linear gradient of acetonitrile (0.1% formic acid)/water (0.1% formic acid) over 90 min at 300 nl/min. Key parameters for the Orbi Elite mass spectrometer were as follows: automatic gain control (AGC), 1 × 10^6^ ions; resolution, 120,000; *m/z* 300 to 1,650; and a top 10 collision-induced dissociation (fragmentation) (CID) method in the ion trap. Key parameters for the QE Plus mass spectrometer were as follows: AGC, 1 × 10^6^ ions; resolution, 70,000; *m/z* 400 to 1,600 with data collected in profile mode and top 10 method. Precursors were selected using a 1.5 *m/z* isolation width, fragmented by higher-energy C-trap dissociation (HCD) with a normalized collision energy of 27 eV. MS/MS scans were acquired at a resolution of 17,500 at 200 *m/z* with an ion target value of 5 × 10^4^, maximum injection time of 50 ms, dynamic exclusion for 15 s, and data collected in centroid mode.

Raw mass-spectrometric data were analyzed using the MaxQuant environment (60), v.1.5.3.30, and employed Andromeda for database search (61) at default settings with a few modifications. The default was used for first search tolerance and main search tolerance. Labels were set to Lys6. MaxQuant was set up to search the reference M. smegmatis proteome (strain MC^2^155) database downloaded from UniProt on 3 February 2015. MaxQuant performed the search for trypsin digestion with up to 2 missed cleavages. Peptide, site, and protein false discovery rate (FDR) were all set to 1% with a minimum of 1 peptide needed for identification but 2 peptides needed to calculate a protein level ratio. The following modifications were used as variable modifications for identifications and included for protein quantification: oxidation of methionine (M), acetylation of the protein N terminus, phosphorylation of serine, threonine, and tyrosine residues (STY), and deamination for asparagine or glutamine (NQ). One unique peptide was required for high-confidence protein identifications, and a minimum ratio count of two peptides (one unique and one razor) were required for SILAC ratio determination. Normalized SILAC ratios (i.e., heavy to light) were used for subsequent analysis.

### Isolation of insoluble protein.

Insoluble proteins were isolated upon DnaK depletion as described in reference 22. Samples were analyzed by immunoblotting for RpoB (BioLegend).

### M. smegmatis DnaK CFU experiments and DnaK immunoblotting.

The DnaK depletion strain was cultured in 7H9 with 25 ng/ml ATC. When they were at mid-log phase, cells were harvested and washed twice with 7H9. The cells were diluted to an OD of 0.02 and separated into three flasks, and 25 ng/ml, 12.5 ng/ml, or 0.78 ng/ml ATC was added. The cultures were grown overnight with shaking. When the OD was ∼1, single-cell suspensions were made by harvesting at 123 × *g* for 8 min without deceleration. The cells were diluted to an OD of 0.01. Cells were diluted and plated for input, and ATC was added back to the original concentration. In a 96-well plate, 200 μl cells was added to RIF at 100 μg/ml, 8918 at 15 μM, or dimethyl sulfoxide (DMSO). After 24 h at 37°C, 10-fold dilutions were made, and cells were plated on 7H11. Numbers of CFU were calculated 3 to 7 days later.

For the DnaK immunoblot, cultures were prepared as described above, except that when cells reached an OD of ∼2.5, 64 OD equivalents were harvested. The cell pellets were washed with PBS/Tween 80 and resuspended in 1 ml PBS/Tween 80. The cells were kept on ice and lysed by bead beating, and lysates were harvested at 18,407 × *g* for 10 min. Supernatant was analyzed by immunoblotting for DnaK as previously described (22).

### FOR experiments.

The DnaK depletion strain was cultured in 7H9 with 25 ng/ml ATC. When they reached mid-log phase, cells were harvested and washed twice with 7H9. The cells were diluted to an OD of 0.01 and separated into two flasks, and 25 ng/ml or 0.78 ng/ml ATC was added. The cultures were grown overnight with shaking. When the OD was ∼1, single-cell suspensions were made by harvesting at 123 × *g* for 8 min without deceleration. The cells were diluted to an OD of 0.2. Cells were diluted and plated for input, and 10^8^ cells were plated on antibiotic (RIF at 240 μM or 8918 at 63 μM)-containing 7H10/ATC. FOR was calculated by dividing the number of resistant colonies by the number of cells plated. Because strains depleted of chaperone have a growth defect, both input and resistant colonies were counted when mutant colonies reached the same size as WT colonies (days 10 to 12). Resistance was confirmed by sequencing *rpoB* from about 10 to 20 colonies from each strain. Note that sometimes (∼1/3 of the time) we saw clumped cultures for low DnaK (0.78 ng/ml ATC) that resulted in low OD upon single-cell suspension. This occurred more when cultures were grown in glass flasks than when they were in plastic flasks. These cultures were discarded.

The other M. smegmatis strains were inoculated from a single colony into 3 ml 7H9 and grown to mid-log phase. This culture was then used to inoculate 30 ml 7H9. When cultures reached an OD_600_ of 0.5 to 0.6, cultures were harvested and resuspended in 3 ml 7H9. Cells were diluted and plated for input, and the remaining cells were plated on 7H10 containing RIF at 240 μM. Plates were incubated at 37°C for 5 days and then counted; incubation was continued until day 10 for a final count, which was used for FOR calculation. FOR was calculated by dividing the number of resistant colonies by the number of cells plated. For strains that produced no colonies on RIF plates, the limit of detection was calculated by using 1/total CFU plated over the experiment (9 cultures added).

### Competition experiments in M. smegmatis.

Ten-milliliter cultures were started in 7H9 in 50-ml conical tubes with appropriate antibiotic selection (kanamycin [20 μg/ml] or SM [20 μg/ml]). After overnight incubation at 37°C, cultures had reached an OD_600_ of 0.4 to 0.6 and were harvested (3,700 × *g*, 10 min, room temperature) and resuspended in 10 ml 7H9 (no antibiotic). For the start of competition experiments, 10 ml of 7H9 in square inkwell bottles was inoculated with the two competing strains each diluted to an OD_600_ of 0.001. For time zero, serial 10-fold dilutions were made in LB_smeg_ and spotted on LB_smeg_ agar with kanamycin or SM selection. Inkwell bottles were incubated at 37°C for 24 h with shaking (150 rpm). At each 24-h time point, cultures were collected and diluted to an OD_600_ of 0.0015 in fresh 7H9, and serial 10-fold dilutions were prepared in LB_smeg_ and spotted on LB_smeg_ with kanamycin or SM. All plates were incubated for 72 h at 37°C, and then CFU were counted. To calculate the competitive index at each time point, the percent CFU for the strain of interest was calculated from total CFU and divided by percent CFU at time zero.

### Doubling-time measurements for M. smegmatis *rpoB*_Mtb_ strains.

Ten-milliliter cultures were started in 7H9 in 30-ml inkwell bottles and grown to an OD_600_ between 0.3 and 0.5. Based on approximated doubling times, cultures were diluted in 10 ml 7H9 and grown for 15 h 37°C with shaking (150 rpm) with a target OD_600_ of ∼0.1. OD_600_ was taken every 1.5 h for up to 9 h. OD_600_ measurements between 0.05 and 0.5 were used to calculate doubling times of each strain. Each strain was prepared in triplicate, and the experiment was repeated 3 times, resulting in 9 calculated doubling times for each strain.

### RpoB immunoprecipitation.

Strains were grown in LB_smeg_ for 15 h at 37°C to an OD_600_ of 0.4. Cultures were cooled on ice and cells were harvested by centrifugation (3,700 × *g*, 10 min, 4°C). All remaining steps were performed at 4°C. Cell pellets were washed in buffer (50 mM Tris, 100 mM NaCl, 10% glycerol, 4 mM EDTA; pH 8.0), and a final resuspension of 500 μl was lysed by bead beating. After bead beating, an additional 500 μl of buffer was added, and the bead/lysate mix was centrifuged at 3,000 × *g* for 5 min. Approximately 1 ml of supernatant was removed for use as the starting lysate. To these lysates, 2.5 μg anti-RNAPβ (BioLegend) was added and incubated for 1 h with shaking. After 1 h, 50 μg of prewashed protein A/G magnetic beads (Pierce) was added and incubated for 1 h. Beads were collected via magnet and washed 4 times with 1 ml buffer with gentle agitation between washes. Elution was done in 100 μl of sample buffer (containing SDS and dithiothreitol [DTT]) heated to 95°C for 5 min prior to the removal of magnetic beads.
