# Supplementary figures and images for: The DnaK Chaperone System Buffers the Fitness Cost of Antibiotic Resistance Mutations in Mycobacteria
Source: mBio. 2021 Mar 30;12(2):e00123-21. doi: 10.1128/mBio.00123-21 (PMC8092207; doi:10.1128/mBio.00123-21)

A.

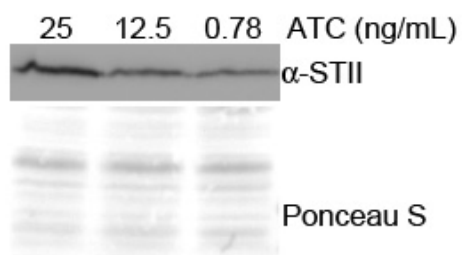

B.

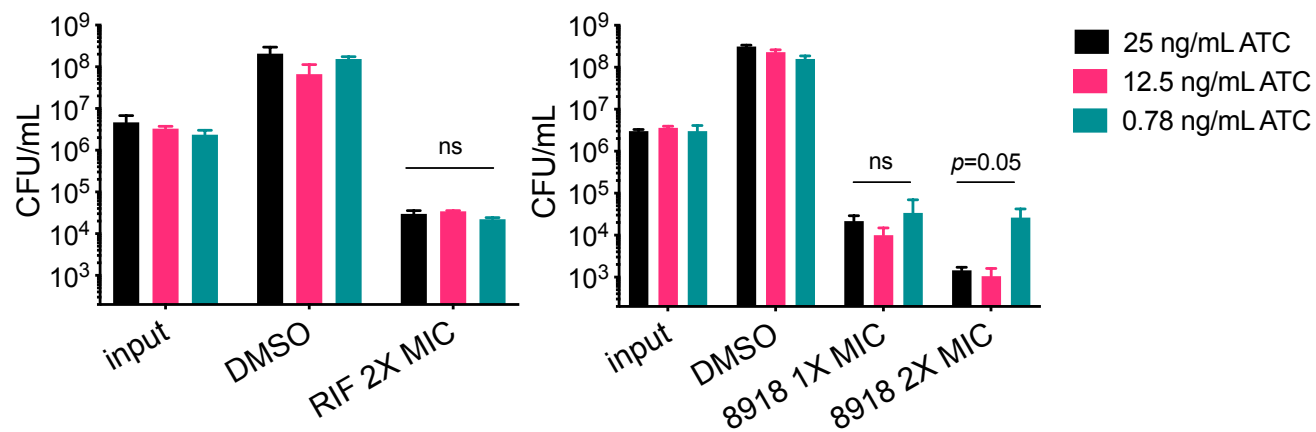

Supplement: FIG S1 [file mBio.00123-21-sf001.pdf]

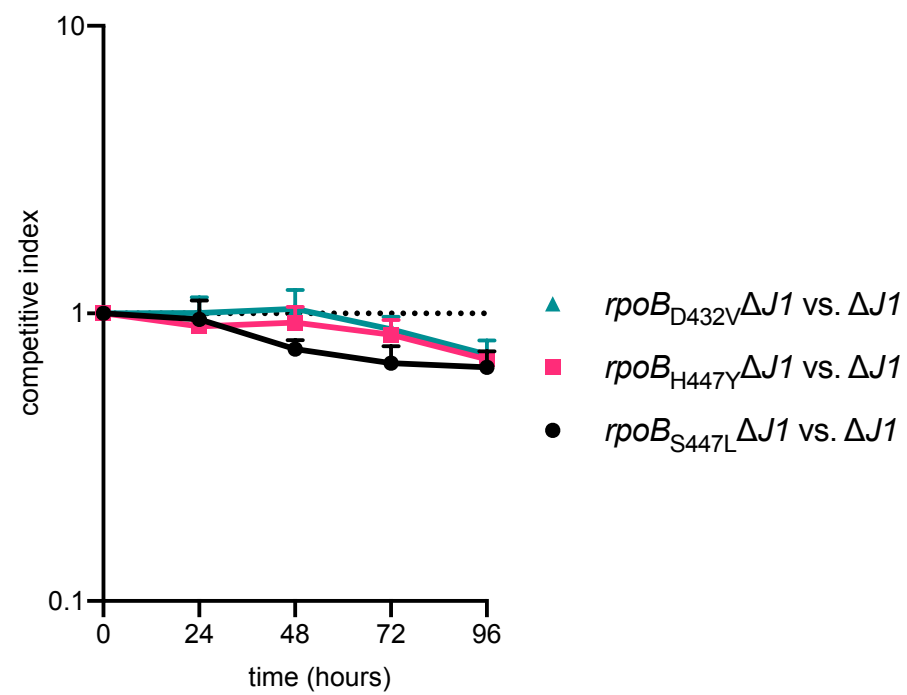

Supplement: FIG S2 [file mBio.00123-21-sf002.pdf]

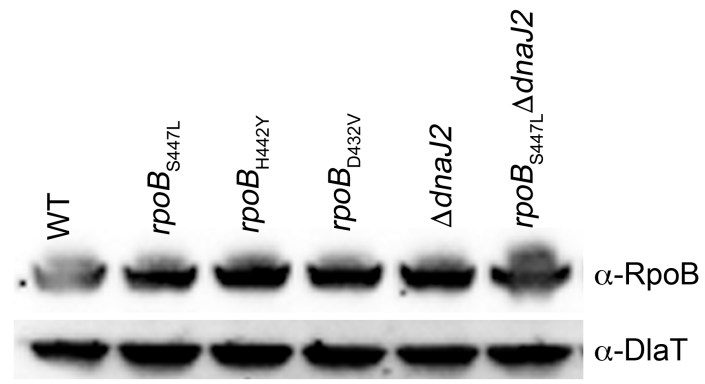

Supplement: FIG S3 [file mBio.00123-21-sf003.pdf]

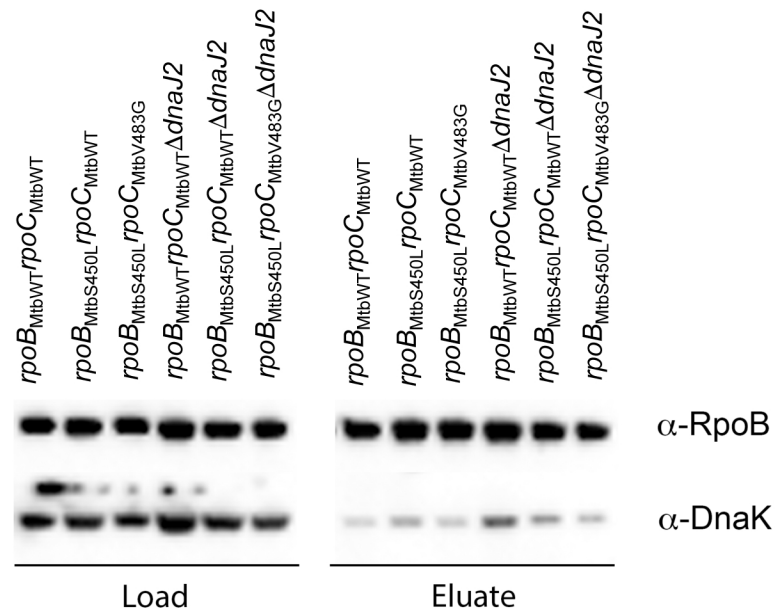

Supplement: FIG S5 [file mBio.00123-21-sf005.pdf]

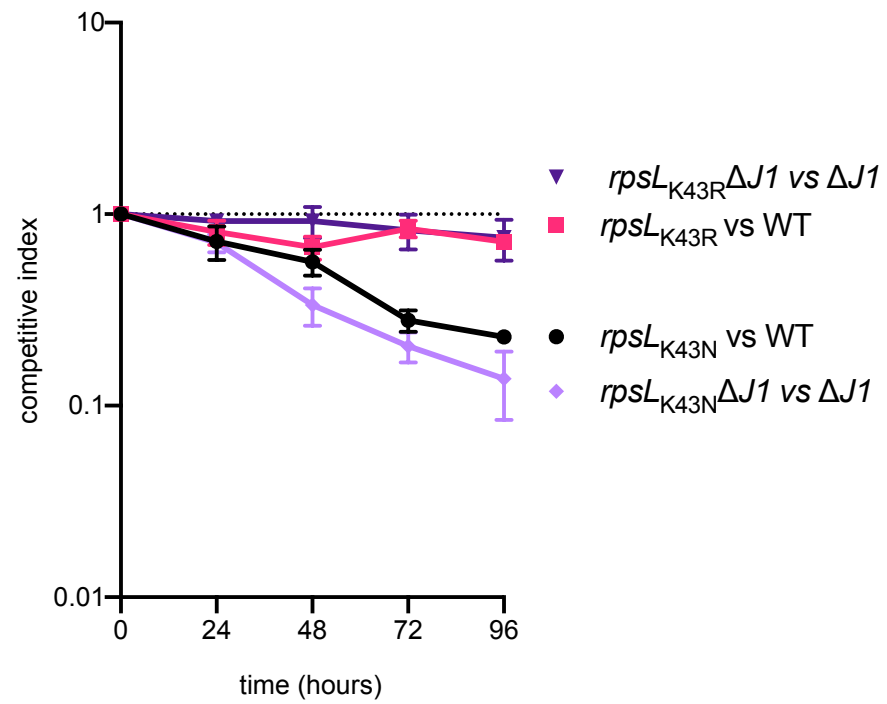

Supplement: FIG S6 [file mBio.00123-21-sf006.pdf]

A.

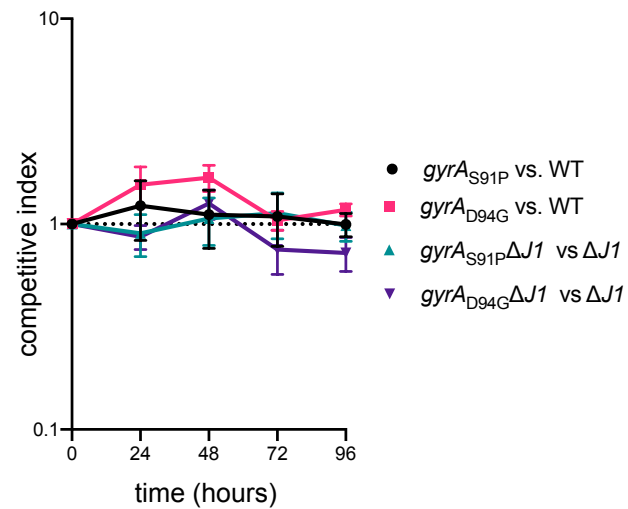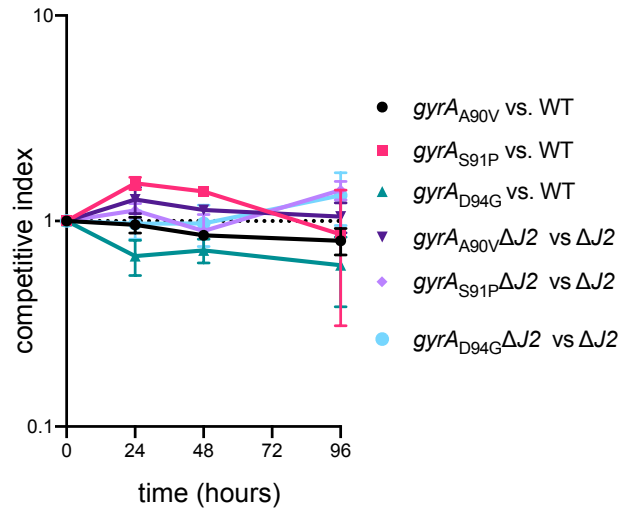

B.

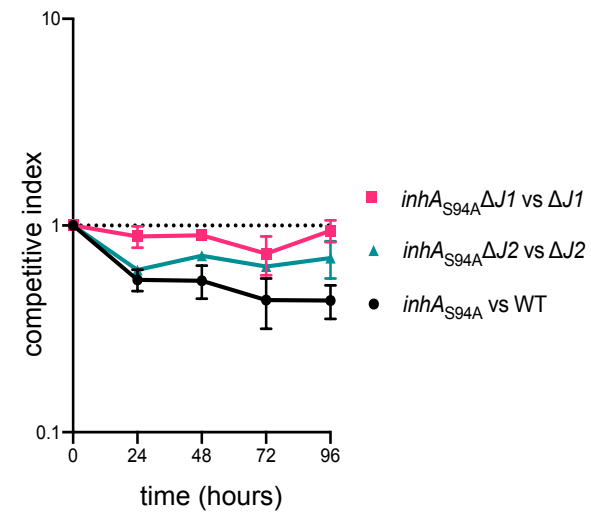

Supplement: FIG S7 [file mBio.00123-21-sf007.pdf]
