# Supplementary material for: The DnaK Chaperone System Buffers the Fitness Cost of Antibiotic Resistance Mutations in Mycobacteria
Source: mBio. 2021 Mar 30;12(2):e00123-21. doi: 10.1128/mBio.00123-21 (PMC8092207; doi:10.1128/mBio.00123-21)
Supplement: FIG S4 [file mBio.00123-21-sf004.pdf]

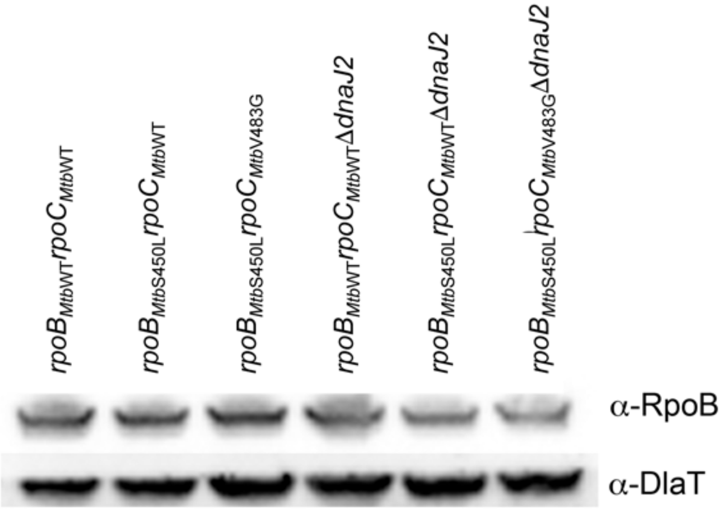

ImageJ quantification relative amount of RpoB over three experiments:

$rpoB_{MtbWT}rpoC_{MtbWT}$	1
$rpoB_{MtbS450L}rpoC_{MtbWT}$	0.97 ± 0.17
$rpoB_{MtbS450L}rpoC_{MtbV483G}$	1.10 ± 0.27
$rpoB_{MtbWT}rpoC_{MtbWT}\Delta dnaJ2$	0.84 ± 0.21
$rpoB_{MtbS450L}rpoC_{MtbWT}\Delta dnaJ2$	0.77 ± 0.35
$rpoB_{MtbS450L}rpoC_{MtbV483G}\Delta dnaJ2$	0.82 ± 0.46
